# Implicit and Explicit Knowledge Both Improve Dual Task Performance in a Continuous Pursuit Tracking Task

**DOI:** 10.3389/fpsyg.2017.02241

**Published:** 2017-12-22

**Authors:** Harald E. Ewolds, Laura Bröker, Rita F. de Oliveira, Markus Raab, Stefan Künzell

**Affiliations:** ^1^Institute of Sports Science, Sports Centre, University of Augsburg, Augsburg, Germany; ^2^Performance Psychology, Institute of Psychology, German Sport University Cologne, Cologne, Germany; ^3^School of Applied Sciences, London South Bank University, London, United Kingdom

**Keywords:** multitasking, implicit motor learning, continuous tracking task, predictability, sequence learning

## Abstract

The goal of this study was to investigate the effect of predictability on dual-task performance in a continuous tracking task. Participants practiced either informed (explicit group) or uninformed (implicit group) about a repeated segment in the curves they had to track. In Experiment 1 participants practices the tracking task only, dual-task performance was assessed after by combining the tracking task with an auditory reaction time task. Results showed both groups learned equally well and tracking performance on a predictable segment in the dual-task condition was better than on random segments. However, reaction times did not benefit from a predictable tracking segment. To investigate the effect of learning under dual-task situation participants in Experiment 2 practiced the tracking task while simultaneously performing the auditory reaction time task. No learning of the repeated segment could be demonstrated for either group during the training blocks, in contrast to the test-block and retention test, where participants performed better on the repeated segment in both dual-task and single-task conditions. Only the explicit group improved from test-block to retention test. As in Experiment 1, reaction times while tracking a predictable segment were no better than reaction times while tracking a random segment. We concluded that predictability has a positive effect only on the predictable task itself possibly because of a task-shielding mechanism. For dual-task training there seems to be an initial negative effect of explicit instructions, possibly because of fatigue, but the advantage of explicit instructions was demonstrated in a retention test. This might be due to the explicit memory system informing or aiding the implicit memory system.

## Introduction

Dual-task studies reveal limitations in human behavior and are therefore an intriguing way to discover the functional properties of the cognitive and motor system. When two tasks are performed simultaneously a decrease in performance is usually observed. Several mechanisms have been proposed to explain this dual-task interference such as bottleneck theories (Welford, 1967; Pashler, 1994; Borst et al., 2010), capacity theories (Kahneman, 1973; Navon and Gopher, 1979; Wickens, 2008), and cross-talk models (Kinsbourne, 1981; Swinnen and Wenderoth, 2004). Bottleneck theories explain dual-task costs by proposing that certain processing stages (response selection and/or response execution) cannot be performed simultaneously. A bottleneck exists so that one task has to finish processing before the other may start, which causes a delay for the second task. Resource theories accept simultaneous processing but state that there is a finite resource (or resources) that put a limit on dual-task performance. Cross talk theories propose that dual-task costs mainly arise when the outcome of one task intervenes with the processing of another (Navon and Miller, 1987). So far these theories have not yielded practical solutions on how to improve dual-task performance (for an overview see Pashler, 1994). When casually observing motor behavior of humans in everyday situations however, it becomes apparent that seemingly successful dual-tasking is a common occurrence: walking down a busy street while talking, or driving a car while listening to the radio for instance. We argue that a key feature of such successful multi-tasking is the predictable nature of at least one of the tasks.

Another feature that theoretically reduces dual-task costs is automatic processing, since it leaves the bottleneck open (Ruthruff et al., 2006b) or frees up limited resources, in order to be able to perform a different task. Neumann (1984) stated that automatic task processing depends on the fulfillment of two demands. According to Neumann there are three sources that specify the parameters that are sufficient to carry out an action: first, procedures stored in long term memory (skills), second, input information from the environment and third attentional mechanisms. As long as skills in conjunction with input information directly specify the parameters of the movement it can be completed without using attentional mechanisms and attentional capacity, and without leading to conscious awareness. Frith and Wolpert (2000) argue that this is exactly how the motor system, equipped with forward models, seems to function. That is, as long as a situation is predictable, for instance going down a familiar set of stairs, and there is no mismatch between expected consequences and results, movements are largely automatic (they occur without awareness or attentional control). Indeed, it would be highly disadvantageous if we were aware of every eye movement or postural adjustment. Therefore, we hypothesize that automaticity and by extension dual-task performance is dependent on the predictability of a task.

One way to make a task predictable is through knowledge, either explicit or implicit. In the current paper implicit knowledge is defined as knowledge shown by performance in the absence of verbalizeable knowledge (Nissen and Bullemer, 1987; Heuer and Schmidtke, 1996). The role of implicit versus explicit knowledge in dual-task situations is controversial. In a review of serial reaction time (SRT) tasks and visuomotor adaptation tasks, Taylor and Ivry (2013) noted that explicit knowledge is mainly used in the planning of action goals while implicit processes are dominant in learning the parameters of movement execution. Although the implicit and explicit knowledge systems can operate in parallel there is evidence that in dual-task conditions only implicit knowledge aids multitask performance (Curran and Keele, 1993). When participants in Curran and Keele’s study were explicitly informed about the sequence in an SRT task, they were much faster compared to non-informed participants, however, when a secondary task was introduced they performed equally to a group that learned the sequence implicitly. Curran and Keele argued that this possibly meant that only the implicit component of knowledge obtained by the informed group was of use in the dual-task situation. The advantage of implicit knowledge has also been demonstrated in sports and motor-related contexts. For instance, novices who learnt a tennis forehand implicitly showed better performance while making complex decisions compared to novices who learnt the forehand explicitly (Masters et al., 2008). In contrast, Blischke et al. (2010) showed that no dual-task costs remained when a key sequence task was learned explicitly and under dual-task conditions. The role of implicit and explicit knowledge in dual-task performance therefore remains unclear. As outlined earlier, we would argue that predictability could be a crucial factor in facilitating optimal dual-task performance, and accepting that implicit and explicit knowledge constitute predictability, both should improve dual-task performance.

Predictability is well-studied in SRT studies which entail simple discrete movements (e.g., Nissen and Bullemer, 1987; Curran and Keele, 1993). Implicit sequence learning is a robust effect found when participants are allowed to practice on this task but equally, performance on the task is easily improved by explicitly pointing out the sequence. In the current study we use a pursuit tracking task that requires continuous movements to track curves which has a less prominent explicit component than the SRT task. The continuous nature of the pursuit tracking task makes it an interesting alternative to the more often used short discrete tasks. It captures performance of real-world tasks such as driving which could be modeled as continuous tracking itself (Raab et al., 2013). The pursuit tracking task requires participants to track a target moving on a screen. The target follows an invisible sinusoidal curve on the screen which consists of three segments (Pew’s paradigm, 1974). To investigate implicit learning, the middle segment remains constant throughout the trials, while the two outer segments vary. It has been demonstrated that this is a reliable manipulation to test for implicit learning, because participant’s performance on the repeating segment is better than on random segments after several practice blocks, even though participants appear not to be aware of the repeating part (Pew, 1974; Wulf and Schmidt, 1997; Zhu et al., 2014; Künzell et al., 2016; de Oliveira et al., 2017).

In Experiment 1 we determined whether a repeated segment within the pursuit tracking task is learned under single task conditions, and if that results in better performance compared to random segments when a second task is introduced (an auditory go/no-go task). We expected better performance and even disappearance of dual-task costs for the repeated segment, which would confirm the hypothesis that tracking of the repeated segment is automatized. Whereas most studies investigating implicit learning in tracking have not tested the effect of explicit knowledge we added this condition to our experiment. Firstly this enables us to investigate the effect of explicit knowledge on a largely motoric task, secondly we are able to test the hypothesis that both types of knowledge would aid dual-task performance since both provide predictability. Experiment 2 was mostly identical to Experiment 1 with the key difference that learning took place under dual-task conditions. This has a practical reason since it can be argued that learning, especially in sports, rarely takes place in single-task conditions. In SRT tasks learning under dual-task conditions is often reduced but not abolished (Frensch et al., 1998). However, there might be a positive effect of a secondary task at later stages in the learning process because attending to well-learned motor skills seems to have a negative effect and this would be diminished in dual-tasking (Beilock et al., 2002). Therefore we may find reduced learning in Experiment 2 but possibly better performance in dual-task conditions compared to single-task conditions after learning.

## Experiment 1

### Materials and Methods

#### Participants

Participants were 37 university students that were divided into two groups: the *implicit group* had 20 participants (*M* = 25.0 years old, *SD* = 2.2) and the *explicit group* had 17 participants (*M* = 25.1 years old, *SD* = 2.8). All participants reported normal or corrected-to-normal vision and no reported neurological disorders. All participants gave informed consent prior to the start of the experiment and received remuneration of 20€ after completing the experiment. The research was approved by the local ethics committee of the University of Augsburg.

#### Experimental Setup

We asked participants to sit at a table in front of a joystick (Speedlink Dark Tornado) and a 24″ computer screen (144 Hz, 1920 × 1080 pixel resolution) which were 40 cm apart. The tracking program ran on a Windows 7 computer and data was recorded at 120 Hz. The stimuli of the auditory go/no-go task were delivered via Sennheiser stereo headphones and we recorded responses with a foot pedal (f-pro USB-foot switch, 9 cm × 5 cm). To ensure that tracking performance was not influenced by moving the joystick through the resting zone, which causes an irregularity in resistance, we made sure that the motion required to position the cursor from the upper to the lower edge of the screen fell within the upper half of the range of motion of the joystick on the y-axis.

#### Tasks and Display

**FIGURE 1:**
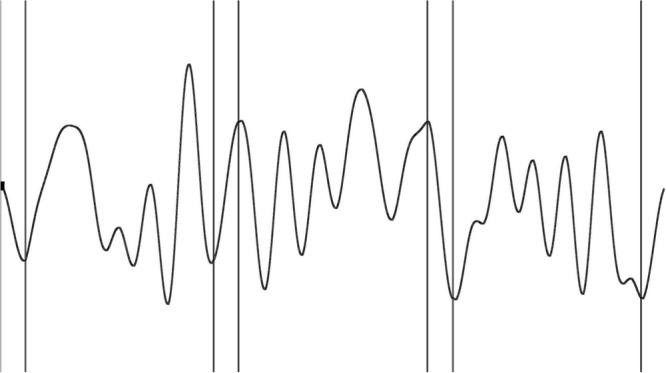
Example of a trial in the tracking task. A trial consists of two random outer segments and a repeating middle segment connected by interpolated segments. Participants tracked a target that moved along the curves, the curves themselves were not visible during the experiment.

The pursuit tracking task was replicated from Künzell et al. (2016). Random tracking segments were created from three segments *j* (left segment), *k* (middle segment) and *l* (right segment), with *j ≠ k, k ≠ l*, and *j ≠ l*. The formula used to create the segments was taken from Wulf and Schmidt (1997):

$$\begin{array}{c} f(x)=b_{0}+\sum_{i=1}^{6}a_{i}\sin(i\cdot x)+b_{i}\cos(i\cdot x) \end{array}$$

with *a_i_ and b_i_* being a randomly generated number ranging from -4 to 4 and *x* in the range [0, 2π]. For this experiment 41 segments of similar length and number of extrema were selected. This is important to guarantee that learning is not attributed to difficulty of the segments (Chambaron et al., 2006). From the 41 segments available, the segment for each participant consisted of a (unique) middle repeated segment and two outer segments selected from the remaining 40, see **Figure 1** for an example. We chose the outer segments in such a way that each segment occurred an equal amount of time across and within participants. This meant that each participant would learn a different middle segment while the overall difficulty level was kept similar. For the tracking task, participants tracked a red target square along the invisible segment by controlling a cursor displayed as a white cross (both target and cursor fit in 19 × 22 pixels). Velocity of the target was constant along the curves, ensuring a uniform difficulty level across the trial. The velocity was the same as in Künzell et al. (2016) because they showed the most effective implicit learning at trial durations between 40 and 44 s.

The secondary task was an auditory go/no-go reaction time task, similar to studies investigating implicit sequence learning in SRT tasks (e.g., Heuer and Schmidtke, 1996). Participants pressed a pedal for high-pitched tones and ignored low-pitched tones (1086 and 217 Hz, 75 ms). On each trial the number of target sounds was 19 or 20 and the number of distractor sounds varied between 13 and 20. The minimum duration between sounds was 1001 ms and no sounds were placed earlier than 500 ms after the start of the trial or 500 ms before the end of the trial.

#### Procedure

After signing the informed consent, participants sat at the table and adjusted their seat and pedal. We tested participants individually. We explained that the cursor and the target moved automatically from left to right along a sinusoidal curve, and the goal was to keep the cursor as accurately as possible on the target by moving the joystick forward and backward (along the x-axis cursor movement was coupled to the target). Every five trials feedback reflecting average performance of the last five trials appeared on the screen.

**FIGURE 2:**
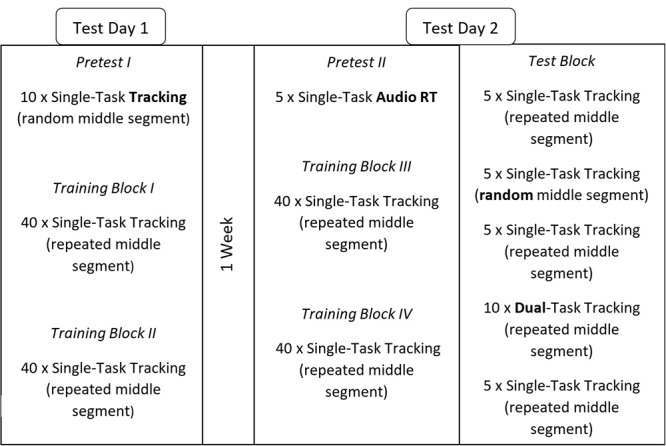
Experimental design for Experiment 1. Both pretests were done for familiarization and stimuli were randomized to prevent learning. The break between training blocks was about a minute. In the test-block, the single-task trials with a random middle segment and the dual-task trials were nested within blocks with trials identical to those of the training blocks to minimize fatigue effects.

On the first day participants completed 10 familiarization trials followed by 10 pre-test trials which were single-task tracking of a random segment. They then completed two training blocks with a repeated middle segment consisting of 40 trials each. Just before the training blocks, participants in the *explicit group* received information that there would be a repeating middle segment in the training blocks (no such instruction was given to the *implicit group*). On the second test day, a week later, participants were prepared for the go/no-go reaction time task by completing five familiarization trials followed by five pre-test trials. They then completed two training blocks as on day 1. At the end of the second test day, participants completed a test-block of 30 trials in different conditions in the following order: five trials as in the training block; five trials with a random middle segment; five trials as in the training block; 10 dual-task trials with the auditory task (participants were asked to pay equal attention to both tasks); five trials as in the training block (see **Figure 2**). After all blocks were completed, the *implicit group* answered a questionnaire to determine how aware they were of the repeated middle segment. The questionnaire contained seven questions designed to gradually probe participants about their knowledge of the repeated middle segment. The questions were: (1) Did you notice anything special during the experiment? (2) Was there something that helped or hindered you while performing the tracking? (3) Did you apply any rules? (4) Did you notice anything special concerning the path of the target? (5) The target followed a certain path. Did you notice any segments in this path? (6) There were three segments in the path, the first, the middle and at the last segment. One of these segments was always repeated? Did you notice? (7) Which segment was the repeated segment, the first, the middle, or the last segment?

#### Data Analyses

The main dependent variable in the tracking task was the root mean square error (RMSE; Wulf and Schmidt, 1997) calculated from the difference between the target curve and the curve made by the user-controlled cursor. We followed the recommendations by Zhu et al. (2014) to take the average performance of the outer segments to compare with the repeated middle segment as they showed that performance deteriorates over time within a trial. For the auditory go/no-go task we recorded reaction times and errors. To test learning effects we submitted average RMSEs to a 4 × 2 × 2 mixed analysis of variance (ANOVA) with within subjects factors Training Block (four training blocks), Segment (middle segment vs. outer segments), and between subjects factors Group (implicit vs. explicit), with a significant Block × Segment interaction indicating learning of the repeating segment. Using the RMSEs from the test-block we checked learning by comparing performance on catch trials (random middle segment) compared to trials with a repeating middle segment. We performed two 2 × 2 × 2 mixed analyses of variance (ANOVA), with within-subjects factors Condition (single-task with repeating segment vs. single-task with random segment in the middle), and Segment (repeated middle segment vs. outer segments), and between-subjects factor Group (implicit vs. explicit). The single-task with repeating segment in the middle condition was the average of the three times we tested this condition, see **Figure 2**. The other 2 × 2 × 2 ANOVA included Condition (single-task vs. dual-task performance, both with a repeating middle segment), Segment and Group. The differences in performance between the repeated segment and the outer segments within the dual-task condition were tested using a paired-samples *t*-test. Finally, to test the effect of the tracking on reaction times (RTs) we performed a 2 × 2 analysis of variance (ANOVA) on reaction times, with factors Task (single or dual) and Group (implicit vs. explicit). A Greenhouse–Geisser correction was used when the assumption of sphericity was violated.

### Results

**FIGURE 3:**
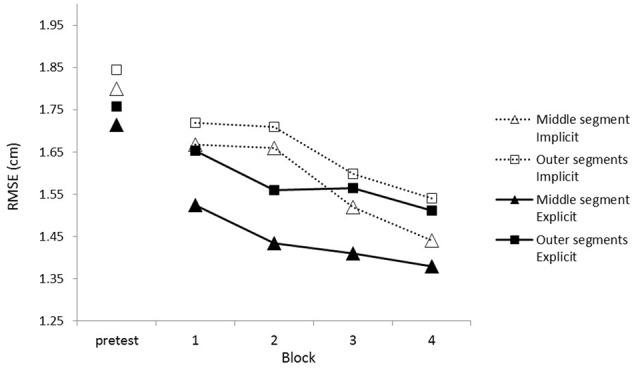
Mean root mean square error (RMSE) scores throughout the training blocks. Training blocks 1–4 had repeating middle segments while the pre-test had random segments in the middle.

First we checked whether the repeated segment was learned at all by analyzing tracking performance during the training sessions. There were overall improvements in tracking indicated by a main effect of Block, *F*(2.22,77.72) = 21.52, *p* < 0.001, $\eta_{p}^{2}$ = 0.381 (see **Figure 3**). Performance was better on the middle segment than on the outer segments as shown by the significant effect of Segment, *F*(1,35) = 45.14, *p* < 0.001, $\eta_{p}^{2}$ = 0.563 (middle *M* = 1.42, *SD* = 0.24; outer *M* = 1.55, *SD* = 0.22). Importantly, a Block × Segment interaction showed that, over the blocks, participants improved more on the repeating middle segment than on the random outer segments, *F*(2.11,73.8) = 7.42, *p* < 0.001, $\eta_{p}^{2}$ = 0.175 (see **Figure 3**). No effect of group was found, *F*(1,35) = 1.99, *p* = 0.168.

**FIGURE 4:**
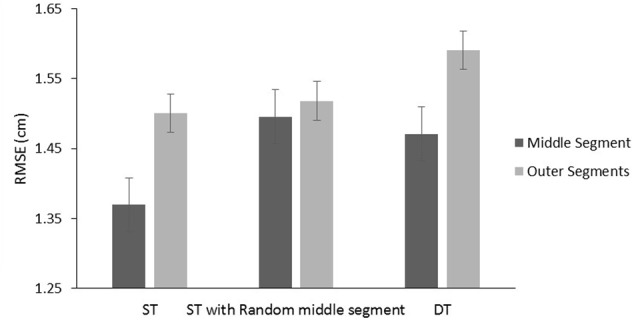
Results of the test-block for the implicit and explicit group together, comparing the effect of putting a random segment in the middle and of dual-tasking with a repeating middle segment. ST, single-task; DT, dual-task.

In order to prove that the repeating middle segment was learned we swapped it for a random middle segment during the test-block. Results revealed that performance for the condition with a repeating middle segment was better than with a random middle segment, *F*(1,35) = 20.13, *p* < 0.001, $\eta_{p}^{2}$ = 0.365, with a Condition (repeating middle segment vs. random middle segment) × Segment (middle vs. outer segments) interaction proving that the difference is due to changes in the middle segment since difference in performance on the outer segments was 0.03 and 0.13 for the middle segment, *F*(1,35) = 20.08, *p* < 0.001, $\eta_{p}^{2}$ = 0.376, see **Figure 4**. An interaction between Condition and Group (implicit vs. explicit) indicated that the difference in performance with a repeating segment in the middle compared to a random segment in the middle was greater for the explicit group than for the implicit group (*M* = 0.18 cm, *SD* = 0.04 for the explicit group, *M* = 0.09 cm, *SD* = 0.03 for the implicit group), *F*(1,35) = 4.17, *p* = 0.049, $\eta_{p}^{2}$ = 0.106.

To test the effect of dual-tasking we compared the single task Condition with a repeated segment in the middle with the dual-tasking, see **Figure 4**. A main effect of Condition (Single-task vs. Dual-task) showed that performance in the dual-task condition deteriorated, *F*(1,35) = 14.13, *p* = 0.001, $\eta_{p}^{2}$ = 0.228. A main effect of Segment indicated better performance on the middle segment, *F*(1,35) = 71.919, *p* < 0.001, $\eta_{p}^{2}$ = 0.673, and a paired samples *t*-test revealed that during dual tasking, performance on the repeated segment (*M* = 1.47, *SD* = 0.23) was better than on the outer segments [*M* = 1.59, *SD* = 0.21; *t*(36) = 6.64, *p* < 0.001]. No main effect of Group could be found, *F*(1,35) < 1, *p* = 0.637, $\eta_{p}^{2}$ = 0.006.

For the second task, the auditory reaction time task, RTs lower than 200 ms and higher than 1000 ms were discarded, resulting in five discarded trials. We found a significant main effect of Condition, *F*(1,33) = 26.78, *p* < 0.001, $\eta_{p}^{2}$ = 0.448, because RTs were significantly slower in the dual-task condition (*M* = 558 ms, *SD* = 58) than in the audio-only pre-test (*M* = 510 ms, *SD* = 57). No effect of Group, *F*(1,33) < 1, *p* = 0.681, and no Condition × Group interaction was found, *F*(1,33) < 1, *p* = 0.551. In another ANOVA no significant effect of Segment, *F*(1,35) = 1.681, *p* = 0.203 could be found, indicating a repeating tracking segment did not lead to better performance on the reaction time task. We did not find a significant Group × Segment effect, *F*(1,35) = 3.636, *p* = 0.065.

Participants of the implicit group could not verbalize explicit knowledge about the repeated middle segment during the first five probing questions. For question 6 two participants said they noticed a repeating segment but for question 7 only one of them correctly identified the middle one as repeating. Answers to question 7, where participants were asked to say which segment was repeating even if they did not notice a repeating segment in question 6, were as follows: 4 said the first segment, 12 said the middle segment, 4 said the last segment.

### Discussion

The purpose of this experiment was to investigate whether predictability helps dual-task performance. Predictability was gained by either implicit or explicit knowledge of the tracking task. Better performance for both groups on the predictable segment during dual-tasking shows that predictability indeed had a beneficial effect on dual-task performance. To the knowledge of the authors this study is the first to use a continuous tracking task to assess the benefit of knowledge gained in single task conditions to performance under dual task conditions. The fact that we found no difference between the explicit and implicit group is in line with SRT task performance under dual-task conditions (Curran and Keele, 1993), which is important because it shows that the implicit and explicit memory system might function similarly for discrete and more continuous tasks. It is often argued that the secondary task prevents the expression of explicit knowledge by using up all attentional resources, meaning the better dual task performance on the repeating segment is due to implicit knowledge only (Nissen and Bullemer, 1987; Curran and Keele, 1993; Heuer and Schmidtke, 1996). The design of the current study does not allow us to determine the contribution of implicit knowledge for the explicit group however.

The implicit group exhibited significantly larger improvements on the repeating middle segment than on the random outer segments and decreases in performance when the repeated segment was exchanged by a random segment, which we take as evidence for implicit learning. Furthermore, only one of the participants revealed explicit knowledge of the repeating segment in the questionnaire, noticing a repeating middle segment and subsequently correctly identifying the middle one. When forced to choose between the three segments, 12 of the 20 participants chose the middle segment. These results are unlike the awareness reported in previous studies (e.g., de Oliveira et al., 2017) and may suggest that participants gained more access to explicit knowledge about the repeating middle segment during the interview than they were aware of during the experiment itself. Another explanation comes from an informal interview after the current study which revealed that participants excluded the first and the last segment being repeated because they remembered that the first segment always started in the middle of the left side of the monitor and then sometimes went up or down. Similarly, the last segment ended by either coming from the top or bottom before ending in the middle at the right side of the monitor. From this they inferred that the middle segment must have been constant. Other authors have suggested that verbal reports might not be the ideal way to assess explicit knowledge in the tracking paradigm since the knowledge is not easily verbalized by its very nature, instead recognition or production of the tracking curve could be a more compatible way of measuring awareness of the repeating middle segment (Chambaron et al., 2006). In any case, the results of the questionnaire do indicate that during the training and test-block participants were unaware of the repeating middle segment.

The explicit group learned the repeating middle segment equally well as the implicit group. This is in contrast with SRT studies which show that knowing the sequence beforehand leads to very fast initial performance (lower RTs) compared to an implicit learning condition (Curran and Keele, 1993). It should be noted that in our study explicit knowledge was gained by instructing participants that the middle segment was always the same, rather than offering knowledge of what the repeating segment looked like beforehand. As such our methods are more in line with Caljouw et al. (2016) who instructed participants to look for the sequence in an SRT task in the explicit condition and found that the younger group, similar in age as the participants in our study, performed comparable to the implicit condition while the older group was worse compared to the implicit condition. The finding that explicit instructions do not benefit motor learning when compared with implicit instructions concurs with findings in whole body movement tracking tasks (Shea et al., 2001) and a catching task on the computer (Green and Flowers, 1991). The design of the current study does not allow for a complete dissociation of implicit and explicit knowledge, therefore it cannot be determined if the positive effect found in the explicit condition in dual-tasking is due to explicit knowledge itself or caused by the implicit learning system being unimpeded by the explicit instructions.

Dual-task costs in the reaction time task were not reduced by predictability of the tracking task. When the tracking task becomes more automatic or less taxing, bottleneck theories predict that processing should become more available for the RT task, either by bypassing the bottleneck (task automatization) or stage-shortening. Resource theories would predict freeing up of resources. Since dual-task costs did not disappear our findings are more in line with the idea of stage-shortening, where the processing stages in the bottleneck model are shortened, rather than automatization (Ruthruff et al., 2006a). However, it is problematic to identify a separate perception, response selection and execution phase in a continuous tracking task, although some authors have tried to do so (Netick and Klapp, 1994). Our findings concur with the results of Heuer and Schmidtke (1996), who did not find an advantage of a learned repeating sequence in an SRT task on the reaction times of a simultaneous go/no-go auditory task with random tones. Further study is needed but it could be that predictability does not influence the mechanisms that produce dual-task interference, rather it improves dual-task performance by facilitating the predictable task only. Since, it could be argued that motor learning rarely takes place in single-task conditions; there usually are distractions or multiple tasks to be performed in many sports for instance, we now turn to the question what happens with implicit and explicit learning under dual-task conditions. Furthermore, since we didn’t find an effect of informing participants about the repeating middle segment for single-task training we need to assess whether this information is beneficial or detrimental in a more demanding learning environment, further clarifying the role of implicit and explicit knowledge.

## Experiment 2

In the second experiment we investigated whether a repeated tracking segment could still be learned under dual-task conditions, depending on whether instructions about the repeating middle segment were given or not. For comparable results we kept the setup and experimental procedure of Experiment 1 but asked participants to perform the training blocks under dual-task condition.

Conflicting results have been found in SRT studies regarding the question of whether implicit learning is still possible in dual-task conditions. Some studies have found acquisition of knowledge is eliminated or severely hampered with a secondary task (Nissen and Bullemer, 1987; Schmidtke and Heuer, 1997). However, Frensch et al. (1998) found that mainly the expression of knowledge is prevented but that implicit learning can still be demonstrated under single-task conditions although, with the same amount of training, the effect was weaker. Blischke et al. (2010) also investigated learning of the SRT with a secondary task. In the training phase this task was combined with a cognitively demanding secondary task and they found dual-task costs completely disappeared. However, since dual task costs appeared again when a different secondary task was used it seems unlikely that the SRT task had been automatized. This was in contrast to a previous study by Blischke (2001), where they found that a ballistic jumping task was completely automatized after dual-task practice. The authors suggested this finding might have been due to the explicit sequential component of both tasks in the SRT study favoring more cognitive control mechanisms (see also Saling and Phillips, 2007). Since the current study uses a task with a stronger motor component rather than an easy to verbalize explicit sequence we expect automatization, shown as an absence of dual-task costs, to be more likely. Furthermore, as learning under dual-task conditions is more resource demanding than single-task training we expect that explicitly informing the participants of the repeating segment might hamper performance, although some authors have suggested that activation of the explicit memory system aids the performance of the implicit system (Reber et al., 1980; Berry and Dienes, 1993). As in the first experiment we do not expect effects of predictability to carry over to the reaction time task, dual-task training would in fact more likely serve to uncouple the two unrelated tasks in order to process them more efficiently, in accordance with the Integrated Task Processing concept of Manzey (1993).

### Materials and Methods

#### Participants

The implicit group contained 19 participants (*M* = 24.0 years old, *SD* = 2.5) and the explicit group had 20 participants (*M* = 23.76 years old, *SD* = 2.44). All participants had normal or corrected-to-normal vision and no reported neurological disorders. All participants gave informed consent prior to the start of the experiment and received remuneration of 20€ or course credit after completing the experiment. The research was approved by the local ethics committee of the University of Augsburg. Experiment setup, task and display were identical to Experiment 1.

#### Procedure

**FIGURE 5:**
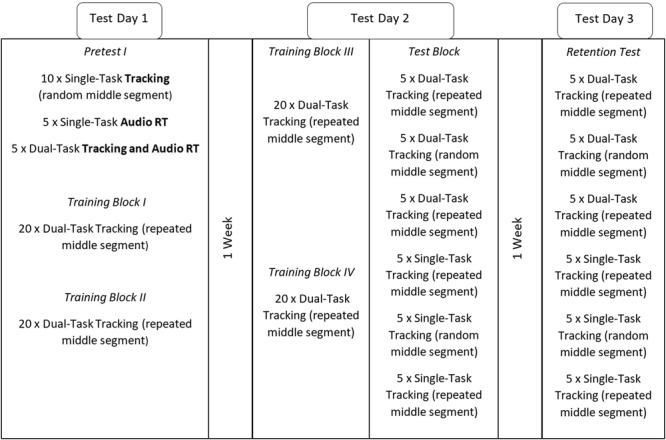
The experimental design of Experiment 2. Note that tracking curves in the pre-test did not contain a repeating segment. The break between training blocks was about a minute. In the test-block and Retention Test, the single-task and dual-task trials with a random middle segment were nested within blocks with trials with a repeating segment to minimize fatigue effects.

The procedure of Experiment 2 differed from Experiment 1 in that participants performed the training of the tracking task always together with the auditory reaction time task (see **Figure 5** for the complete protocol). The pre-test included single task and dual-task trials. Participants were asked to try their best on both tasks equally throughout the experiment. Another difference with Experiment 1 is that the training blocks contained 20 trials instead of the 40 trials because we found in a pilot that fatigue played a much larger role in the dual-task training than the single task training. Furthermore, the test-block was expanded to contain both testing under single and dual task conditions. Lastly, a retention test was done on a third day, a week after the test-block was performed. The retention test was exactly the same as the test-block and was added to see if learning was consolidated and test performance without the possibly confounding effect of fatigue resulting from putting the test-block at the end of multiple training blocks.

#### Data Analyses

**FIGURE 6:**
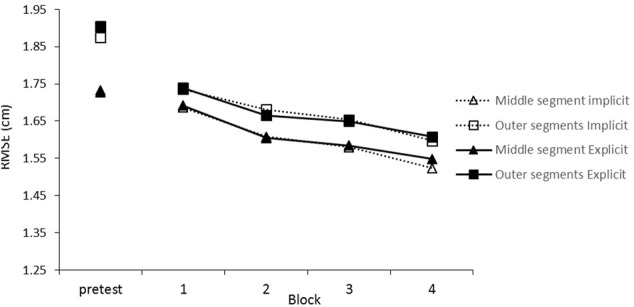
Root mean square error scores for the training blocks. The pre-test, Block 1 and Block 2 were completed on 1 day, Block 3 and Block 4 were completed on another day.

To test learning effects during the training blocks we submitted RMSE scores to a 4 × 2 × 2 mixed analysis of variance (ANOVA) with within subjects factors Training Block (four training blocks), Segment (repeated middle segment vs. outer segments), and between subjects factors Group (implicit vs. explicit). To analyze test-block and retention test performance on a learned middle segment against performance on a random segment for dual or single-task conditions we had the choice to either compare the repeated middle segment with a random middle segment or to compare the repeated middle segment with the random outer segments. Since the data suggested that segment position might be a confounder, with better scores on the middle segment during the pre-test (see **Figure 6**), we chose the first option and analyzed RMSE scores with a 2 × 2 × 2 × 2 ANOVA with within-subjects factors Test (test-block vs. retention test), Segment (Constant vs. Random, both in the middle), Condition (Single-task vs. Dual-task) and between-subjects factor Group (Implicit vs. Explicit). Similarly we submitted reaction times to a 2 × 2 × 2 ANOVA with within-subjects factors Test, Condition (Repeating segment in the middle vs. Random segment in the middle) and Group. To check for the existence of dual-task costs during the test-block and retention test we performed another 2 × 2 × 2 ANOVA with within-subjects factors Test (Test-block vs. Retention test), Condition (Dual-task with repeating segment in the middle vs. Single-task) and Group.

### Results

The questionnaires revealed that one participant in the implicit group discovered the repeating middle segment, this data was removed from analyses.

During the training blocks participants improved, *F*(1.57,58.05) = 7.21, *p* = 0.003, $\eta_{p}^{2}$ = 0.16, and performance on the repeated segment was better than on the random segments, *F*(1,37) = 11.45, *p* = 0.002, $\eta_{p}^{2}$ = 0.24, but crucially we could not demonstrate an interaction effect between Block and Segment, *F*(2.19,80.98) < 1, *p* = 0.672, indicating that learning of the repeating segment was not better than learning of the random segments, see **Figure 6**. No difference between the implicit and explicit group could be found, *F*(1,37) < 1, *p* = 0.972.

**FIGURE 7:**
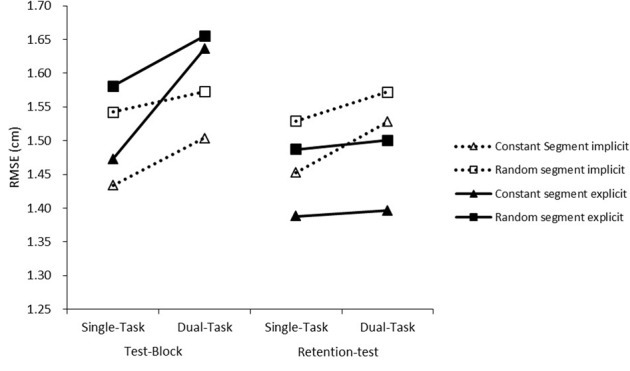
Root mean square error scores for the test-block and the retention test performed 1 week later.

In the test-block and retention test, see **Figure 7**, we found better tracking of a constant segment, *F*(1,36) = 10.61, *p* = 0.002, $\eta_{p}^{2}$ = 0.228. No significant dual-task costs could be found although it almost reached significance, *F*(1,36) = 3.36, *p* = 0.075. We did not find a significant interaction between Condition (dual-task vs. single-task) and Segment (constant vs. random), *F*(1,36) = 1.65, *p* = 0.207. No difference between the implicit and explicit group could be found, *F*(1,36) < 1, *p* = 0.97. There was a significant interaction effect of Test and Group (test-block vs. retention test), *F*(1,36) = 4.21, *p* < 0.048, $\eta_{p}^{2}$ = 0.11, indicating that the explicit group improved from test-block to retention-test while the implicit group did not.

No difference in reaction times between the repeating segment (*M* = 538 ms, *SD* = 69) and random segment was found (*M* = 538 ms, *SD* = 72), *F*(1,36) = 3.28, *p* = 0.083, nor was there a difference between the implicit (*M* = 531 ms, *SD* = 69) and explicit group (*M* = 554 ms, *SD* = 73), *F*(1,37) = 1.39, *p* = 0.246. We did find better performance on the retention-test (*M* = 527 ms, *SD* = 66) compared to the test-block performed earlier (*M* = 557 ms, *SD* = 76), *F*(1,36) = 16.31, *p* < 0.001, $\eta_{p}^{2}$ = 0.312. Dual-task costs were still present at the test-block and retention test when comparing dual-task performance on the repeated segment (*M* = 538 ms, *SD* = 69) with single task performance (*M* = 482 ms, *SD* = 57), *F*(1,36) = 57.19, *p* < 0.001, $\eta_{p}^{2}$ = 0.614. Moreover, a significant interaction effect between Condition and Group, *F*(1,36) = 5.90, *p* = 0.020, $\eta_{p}^{2}$ = 0.141, indicated that the difference in reaction times between Dual-task with a repeating segment and Single-task was greater for the explicit group (*M* = 76 ms, *SE* = 8) than the implicit group (*M* = 39 ms, *SE* = 13).

### Discussion

For the second experiment we did not find learning due to repetition of the repeated middle segment during the training blocks, but we did see better performance on a repeated middle segment compared to the random middle segment during the test-block. These results concur with Frensch et al. (1998) in that a secondary task does not prevent learning, rather the expression of what is learned is suppressed. Although not significant, there seems to be some indication that explicit instructions hamper performance during dual-tasking more than no instructions, see **Figure 7**. This raises the question what the content of the learned information was for the explicit group. In the current experiment we cannot say whether the explicit group made use of explicit knowledge or that for them implicit knowledge was also helpful, whereas the interviews clearly prove that the implicit group did not make use of explicit knowledge. In other words, the results for the explicit group are consistent with the view that explicit knowledge is helpful for learning but the expression is suppressed during dual-tasking. But the results also concur with the view that only *implicit* learning occurs under dual-task conditions and that the explicit group in the current study acquired implicit knowledge in addition to the in dual-task situations harmful explicit knowledge.

The explicit group improved their tracking performance from the test-block to the retention test seemingly beyond that of the implicit group, whose performance remained the same. There is some evidence that the explicit memory system might inform or stimulate the implicit learning system (Reber et al., 1980; Willingham, 1999), although the contrasting view that explicit knowledge, especially instruction on how to perform movements, is also often found to be detrimental to the formation of motor skills (Poldrack and Packard, 2003). Our results are compatible with both these views since we did not give explicit instructions on how to perform the tracking movements, rather the explicit instructions more likely had the effect of focusing attention to the repeating segment aiding implicit learning.

As in the Experiment 1 reaction times did not decrease during the predictable tracking segment, possibly a sign of effective task shielding, a concept closely related to the Integrated Task Processing concept of Manzey (1993) introduced earlier, which states that training two tasks together should enable participants to uncouple them, therefore reducing interference and improving dual-task performance. Task shielding is useful to protect a primary task from distractors but might also lead to less cognitive flexibility, so that the predictability of the tracking task in our study could not be exploited for the reaction time task (Plessow et al., 2011, 2012). If the strategy during the current experiment was to decouple the tasks there is no reason to assume that predictability of one task influences performance on the other task. The influence the two tasks might have on each other, for better or worse, is exactly what participants learned to avoid. Another explanation is that predictability does not transfer between modalities, in line with the idea of multiple resources. The visual-manual system may not share resources with the auditory-pedal system and a reduction of resource usage for predictability does not help the other system.

## General Discussion

The finding of both experiments suggests there is a beneficial but limited role of predictability in multitasking performance. Our task differs from the SRT task used in similar investigation but there seems to be converging evidence that in dual-task situations explicit knowledge of a sequence is not as beneficial as implicitly learned movement sequences (Heuer and Schmidtke, 1996; Frensch et al., 1998). Although the effect was not statistically significant, our results agree with this: after single-task training both explicitly instructed and implicitly trained participants performed better on predictable segments of the tracking segment whereas after dual-task training, *initially* only the implicit group demonstrated learning effects in the dual-task condition. However, when tested again a week later the explicit group demonstrated similar learning effects and a larger overall improvement in performance compared to the implicit group. A possible explanation is that explicit instructions aid implicit motor learning but initially interfere with the expression of knowledge. Another explanation is that explicit instructions fatigued the participants more, the test-block was performed after two training blocks while the retention test was performed on a different day without any training blocks.

The fact that we found learning after dual-task training is in contrast with the hypothesis of Nissen and Bullemer (1987) who argued that learning may occur without awareness but always requires attention, following from their findings that no learning was found when combining the SRT task with a secondary task. Since then this view has been sharpened by results from Cohen et al. (1990) and Curran and Keele (1993) who found evidence that unique sequences, where each item is always uniquely followed by a certain other item, can be learned in the presence of attentional distraction, whereas sequences that lacked such an item to item connection could not. As such our findings are in agreement with the idea of a non-attentional and an attentional learning system, either with or without awareness.

A limitation of the current study is that while we tested for the absence of explicit knowledge in the implicit group we did not confirm the existence of explicit knowledge in explicit group. Future studies should employ methods to test how explicit knowledge of the repeating segment is stored, reproducing or identifying the repeating segment might be more suitable methods of assessing explicit knowledge than describing the curve. Furthermore, a comparison with an implicit group would be necessary because these methods cannot completely distinguish between implicit and explicit knowledge (Chambaron et al., 2006).

## Conclusion

Predictability through knowledge aids dual-task performance, which can be explained by different learning mechanisms. In dual-task training explicit instructions seem to initially worsen performance, possibly because of fatigue, but ultimately they lead to better consolidation of motor learning. The other main finding is that predictability of one task does not increase performance in the other task. Future research will focus on further elucidating the role of predictability in dual-task performance by investigating the effect of making each task predictable, for instance making the auditory reaction time task be a constant sequence, or by making both tasks predictable as a unit, facilitating task integration and countering task-shielding. The latter avenue of research is intriguing because it challenges us to think about what a ‘task’ is: can performing two integrated tasks still be seen as dual-tasking (Künzell et al., 2017). Although difficult to access and likely dependent on individual differences, it may be possible to present task boundaries in such a way that the manner in which two tasks are conceptualized facilitates multitasking performance, possibly through manipulation of instructions or feedback (Dreisbach et al., 2007; Freedberg et al., 2014; Bröker et al., 2017).

## Ethics Statement

This study was carried out in accordance with the recommendations of the ethical guidelines of the ethics committee of the University of Augsburg. All subjects gave written informed consent in accordance with the Declaration of Helsinki. The protocol was approved by the Augsburg University.

## Author Contributions

All authors listed have made a substantial, direct and intellectual contribution to the work, and approved it for publication.

## Conflict of Interest Statement

The authors declare that the research was conducted in the absence of any commercial or financial relationships that could be construed as a potential conflict of interest.
